# Draft Genome Sequence of New Vancomycin-Resistant Enterococcus faecium Sequence Type 1421

**DOI:** 10.1128/genomeA.00438-18

**Published:** 2018-05-17

**Authors:** Kelvin W. C. Leong, Louise A. Cooley, Ronan F. O’Toole

**Affiliations:** a School of Medicine, College of Health and Medicine, University of Tasmania, Hobart, Tasmania, Australia; b Royal Hobart Hospital, Hobart, Tasmania, Australia; c Department of Clinical Microbiology, School of Medicine, Trinity College Dublin, St. James's Hospital, Dublin, Ireland

## Abstract

The spread of vancomycin-resistant Enterococcus faecium (VREfm) has become a challenge to health care infection control worldwide. In 2015, a marked increase in VREfm isolation was detected in acute public hospitals in Tasmania. We report here the draft whole-genome sequence of a newly designated VREfm sequence type, sequence type 1421 (ST1421).

## GENOME ANNOUNCEMENT

Health care-associated infections (HAIs) are frequent adverse events for patients receiving care and can result in prolonged hospital stay, recurrent illness, and higher financial costs for health systems. The World Health Organization has estimated the pooled prevalence of HAIs in low- and middle-income countries to range from 5.7% to 19.1% (1). In Tasmania, approximately 2,200 HAI cases occur each year among admitted hospital patients (2). The leading causes of HAIs in the state are Enterococcus faecium, Staphylococcus aureus, and Clostridium difficile (3). While the incidence of health care-associated bacteremias due to Staphylococcus aureus, a possible sentinel for hand hygiene compliance (4), has remained relatively stable in Tasmania in recent years, notifications of vancomycin-resistant E. faecium (VREfm) have increased significantly in the state’s acute public hospitals since the beginning of 2015 (3).

The genotyping of VREfm isolates is important for monitoring the spread of VREfm between different regions or hospitals. Multilocus sequence typing is widely used to type E. faecium, but in some instances, isolates may be nontypeable due to the absence of one or more of the seven loci. This limits the surveillance of this subset of VREfm infections. Here, genomic DNA of a Tasmanian isolate of a nontypeable strain, TASVRE1, was sequenced using an Illumina MiSeq instrument. A total of 1,175,634 paired-end reads was mapped to the publicly-available complete reference genome sequence of E. faecium sequence type 18 (ST18) DO (TX16) (GenBank accession number NC_017960) (5) using Snippy (https://github.com/tseemann/snippy). This yielded an average read depth of 49.6-fold, covering 96.4% of the reference genome. Gubbins (https://github.com/sanger-pathogens/Gubbins) was used with the FASTA alignment file as input for the prediction and extraction of regions of recombination as previously described (6). A total of 2,874 variant sites were identified relative to the E. faecium ST18 DO genome and consisted of 2,339 single-nucleotide variants (SNVs), 357 complex-nucleotide variants and 187 insertions/deletions. A 2,618,166-bp draft genome was assembled *de novo* using the SPAdes assembler (v3.10.0) (7) and ordered with respect to the E. faecium DO genome using the Algorithm Based Automatic Contiguation of Assembled Sequences (ABACAS) (8), generating 245 contigs (>500 bp).

Using the ResFinder antibiotic resistance gene analysis tool (https://cge.cbs.dtu.dk/services/ResFinder/) at the Center for Genomic Epidemiology (CGE) database, the *vanA* vancomycin resistance locus was detected in TASVRE1. Using the Velvet plugin in Geneious (Biomatters Ltd.) (5), the raw sequence read pairs were assembled *de novo* with the Velvet optimizer to automatically determine the *k*-mer length in the range of 27 to 35 to optimize genome assembly. The genome assembly was checked using Geneious to locate members of the seven multilocus sequence type (MLST) housekeeping genes. From the whole-genome data for TASVRE1, it was possible to establish the complete absence of the *pstS* gene, as well as upstream coding sequences for three hypothetical proteins. Upon provision of the draft genome assembly of TASVRE1 to the curators of the PubMLST database (https://pubmlst.org/efaecium/), the lack of the *pstS* gene was verified and the strain was assigned to the recently designated ST1421. This study highlights the utility of whole-genome sequencing in deciphering the genotypes of bacterial isolates that are nontypeable with conventional techniques.

### Accession number(s).

This whole-genome shotgun project has been deposited at DDBJ/ENA/GenBank under the accession number OOIM00000000. The version described in this paper is version OOIM01000000.
